# Exploration of the underlying comorbidity mechanism in psoriasis and periodontitis: a bioinformatics analysis

**DOI:** 10.1186/s41065-023-00266-z

**Published:** 2023-02-10

**Authors:** Hao Lei, Xin Chen, Ziyang Wang, Zixuan Xing, Wenqian Du, Ruimin Bai, Ke He, Wen Zhang, Yan Wang, Yan Zheng

**Affiliations:** 1 Department of Dermatology, the First Affiliated Hospital of Xi’an Jiaotong University, Xi’an, 710061 China; 2 State Key Laboratory of Military Stomatology & National Clinical Research Center for Oral Diseases & Shaanxi Clinical Research Center for Oral Diseases, Department of Orthodontics, School of Stomatology, The Fourth Military Medical University, Xi’an, 710032 China; 3 Department of Medicine, Xi’an Jiaotong University, Xi’an, 710061 China

**Keywords:** Psoriasis, Periodontitis, Differentially expressed genes (DEGs), Comorbidity, Bioinformatics

## Abstract

**Background:**

Increasing evidence indicates that psoriasis (PSO) and periodontitis (PD) are likely to occur together, however, the underlying mechanism remains unclear.

**Materials and methods:**

The expression profiles of PSO (lesion vs non-lesion, GSE30999, GSE14905) and PD (affected vs unaffected gingival tissue, GSE16134, GSE10334) were downloaded from the GEO database. First, we investigated the common differentially expressed genes (DEGs) of PSO and PD. Then, GO and KEGG enrichment analysis, protein interaction network (PPI) construction, and hub gene identification analysis were carried out. Finally, GO and KEGG enrichment analysis, miRNA interaction analysis, and transcription factors (TFs) interaction analysis for hub genes were performed.

**Results:**

Eighteen DEGs were identified for further analysis, including 15 up-regulated genes and 3 down-regulated genes. 9 hub genes were then identified via Cytohubba, including IL1B, CXCL1, CXCL8, MMP12, CCL18, SELL, CXCL13, FCGR3B, and SELE. Their functions are mainly enriched in two aspects: neutrophil chemotaxis and migration, chemokine activation and interaction. The enriched signaling pathways includes three categories: host defense, inflammation-related signaling pathways, and disease-related pathways. 9 common miRNAs based on experimental evidence and 10 common TFs were further identified in both PSO and PD.

**Conclusion:**

Our study revealed possible comorbidity mechanisms in PSO and PD from the perspective of bioinformatics tentatively. The data can present new insight for joint prevention and treatment of in PSO and PD, as well as provide data support for further prospective studies.

**Supplementary Information:**

The online version contains supplementary material available at 10.1186/s41065-023-00266-z.

## Introduction

Psoriasis (PSO) is an immune-mediated inflammatory skin disease that affects more than 60 million adults and children worldwide [1]. It’s defined by abnormal and rapid keratinocyte differentiation and thickened epidermis [2]. Several systemic disorders are associated with it, including obesity, hypertension, hyperlipidemia, diabetes, metabolic syndrome, heart disease, etc. [3]. Streptococcal infection, trauma, certain medications, smoking, abuse of alcohol and stress all contribute to PSO [4]. It is important to note that the pathogenesis of PSO is highly complex, involving both classical immune cells and tissue cells. By producing cytokines such as TNF-α, IFN-γ, IL-17, and IL-22, these cells promote epidermal hyperproliferation and the production of numerous antimicrobial proteins, growth factors, and chemokines [5]. A current understanding holds that the IL-17/23 signaling axis is a dominant role of PSO occurrence and development [6].

Periodontitis (PD), affecting roughly 10%-15% people worldwide, is a chronic and destructive periodontal disease that results from several dynamic interactions [7]. Inflammation of the periodontal tissue and resorption of alveolar bone is an important feature of this disease [8]. Numerous chronic diseases are associated with it, including cardiovascular disease, Alzheimer's disease, Diabetes, Rheumatoid arthritis, etc. [9]. Both periodontal bacteria and host immunity are involved in the occurrence and development of PD [10]. A pathogenic complex of Porphyromonas gingivalis, Treponema dentate, and Forsythia was once thought to play a significant role in the occurrence of PD based on isolation and culture studies. With the development of immunological research, it has become increasingly clear that local host immunity plays a vital role. Furthermore, a complex regulatory network comprised of cytokines and inflammatory cells explains the connection between PD and its complications, including IL-1, IL-6, IL-17, TNF, Th17 cell and Treg cell-related cytokines [11].

The above contents suggest that both PSO and PD are related to amounts of systemic diseases and inflammatory response. Meanwhile, more and more evidence indicates that there is a bidirectional association between PSO and PD from the perspective of clinical research. Several observational researches have shown that individuals with PSO have significantly more tooth loss, more plaque and bleeding sites on probing, and a significantly higher prevalence of PD than controls [12–14]. Researchers found that poor living habits such as smoking and drinking interfered with the correlation between patients with PSO and PD in univariate analysis. When confounding factors were removed from the analysis, the hazard ratio between PSO and PD was reduced, but remained statistically significant [15, 16]. What's more, some cohort studies have emphasized this bidirectional association. In PSO, after 15 years of follow-up, the research showed an overall increased risk of new PD in patients with PSO, such as increased risk of PD in mild PSO (IRR = 1.66), severe PSO (IRR = 2.24) and psoriatic arthritis (IRR = 3.48) compared with the control [17].In PD, a prospective study noted an increased risk of PSO in PD patients (HR = 1.52, 95%CI = 1.38–1.70) after 5-years follow-up [18].Another study with a large sample found that there is a significantly higher risk of PSO in PD patients compared to non-PD patients (HR = 1.116, 95%CI = 1.101–1.13) over 9 years of follow-up [19]. Contrary to what is generally believed, one literature reported that the periodontal debris index, calculus index, and plaque index were comparable between PSO and control, but there was no statistically difference [20]. Nonetheless, multiple systematic reviews and meta-analyses point to a strong association between PSO and PD [21–23].

Despite the strong clinical correlation between PSO and PD, the underlying mechanisms remain unclear. Based on previous studies, we speculated that there are similar mechanisms in the pathogenesis of PSO and PD, such as some common DEGs and pathways. To verify this hypothesis, Bioinformatics is an ideal approach and perspective. It’s a tool designed to understand biological phenomena using information science and statistical methods, which is widely used in deep sequencing, imaging and mass spectrometry analysis, and plays an important role in the study of molecular mechanisms such as tumors, systemic diseases and rare diseases [24].

Hence, the purpose of this study is to understand the correlation between PSO and PD, and explore the underlying comorbidity mechanism through bioinformatics analysis. So as to provide certain data support and breakthrough point for the joint prevention and treatment of PSO and PD.

## Results

### Information of GEO datasets

Based on the criteria we established before, GSE30999, GSE14905, GSE16134 and GSE10334 were obtained. The information of these datasets was shown in (Supplementary Table 1), including the disease types, platforms, sample types, and quantities.

### Identification of common DEGs

**Fig. 1:**
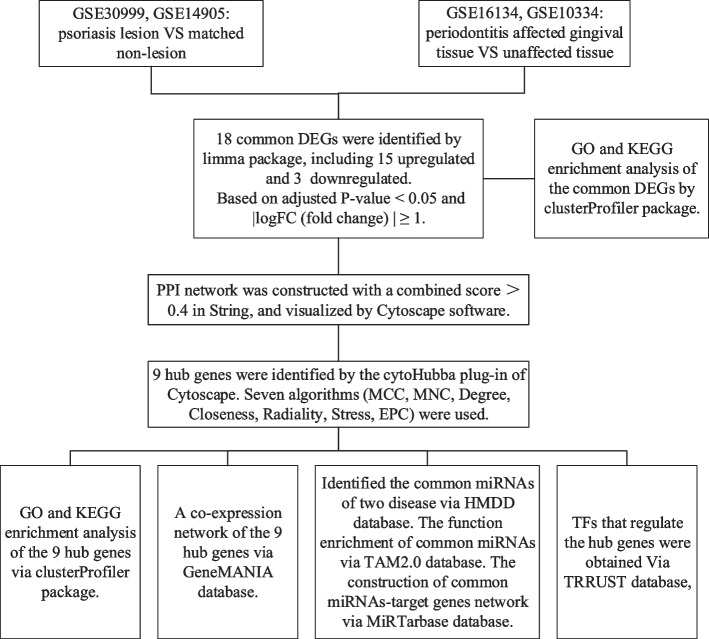
The flowchart of this study

**Fig. 2:**
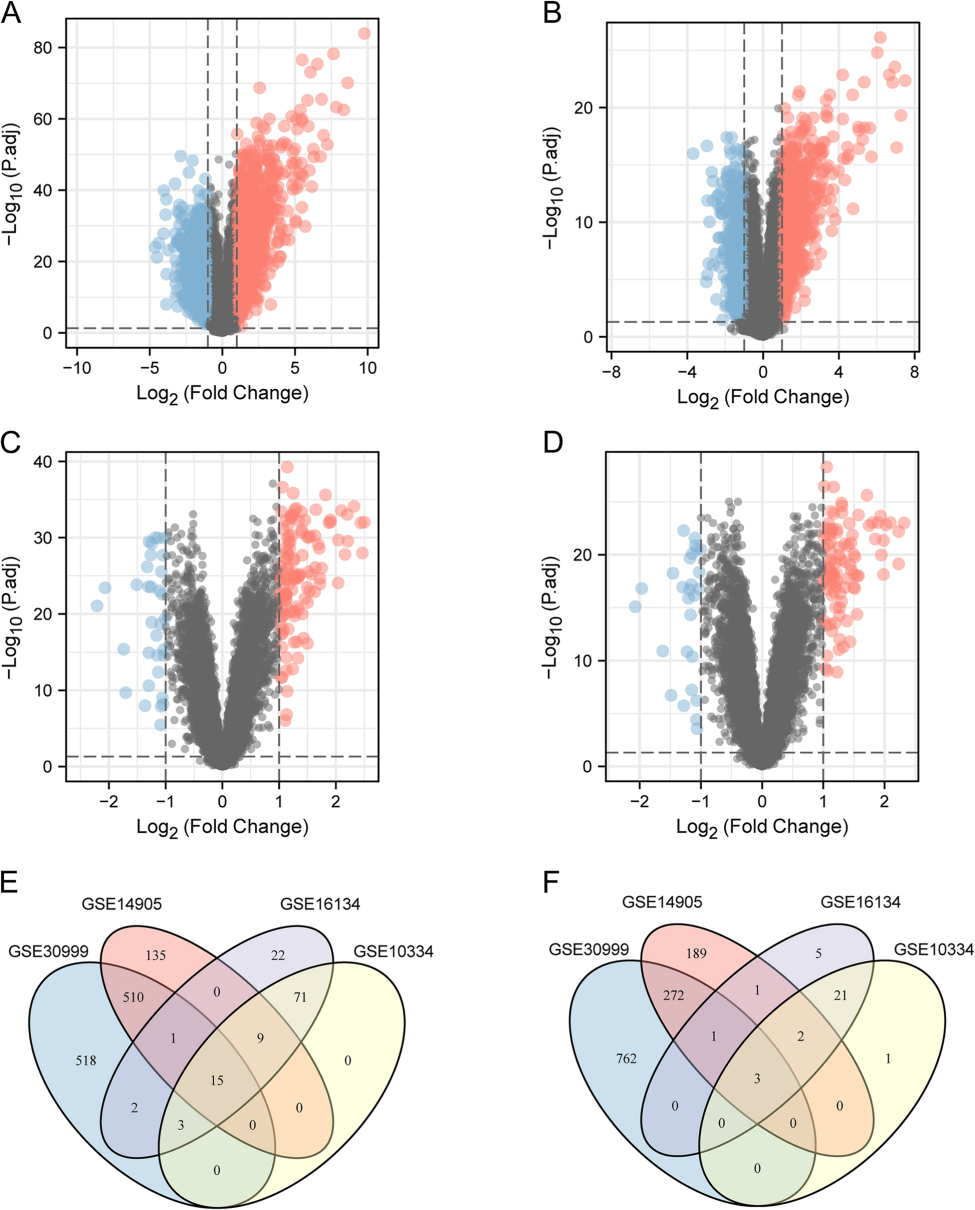
Volcano and Venn diagram. **A**. The volcano map of GSE30999. **B**. The volcano map of GSE14905. **C**. The volcano map of GSE16134. **D**. The volcano map of GSE10334. Red represents upregulated genes, and blue represents downregulated genes. **E.** The four datasets have an overlap of 15 common upregulated genes. **F**. The four datasets have an overlap of 3 common downregulated genes

As shown in the flowchart (Fig. 1),there were 2087 DEGs obtained in GSE30999, 1138 in GSE14905, 156 in GSE16134 and 125 in GSE10334(Fig. 2A,B,C,D). A Venn diagram intersection revealed 15 common upregulated DEGs and 3 common downregulated DEGs (Fig. 2E, F).

### Enrichment analysis of common DEGs

**Fig. 3:**
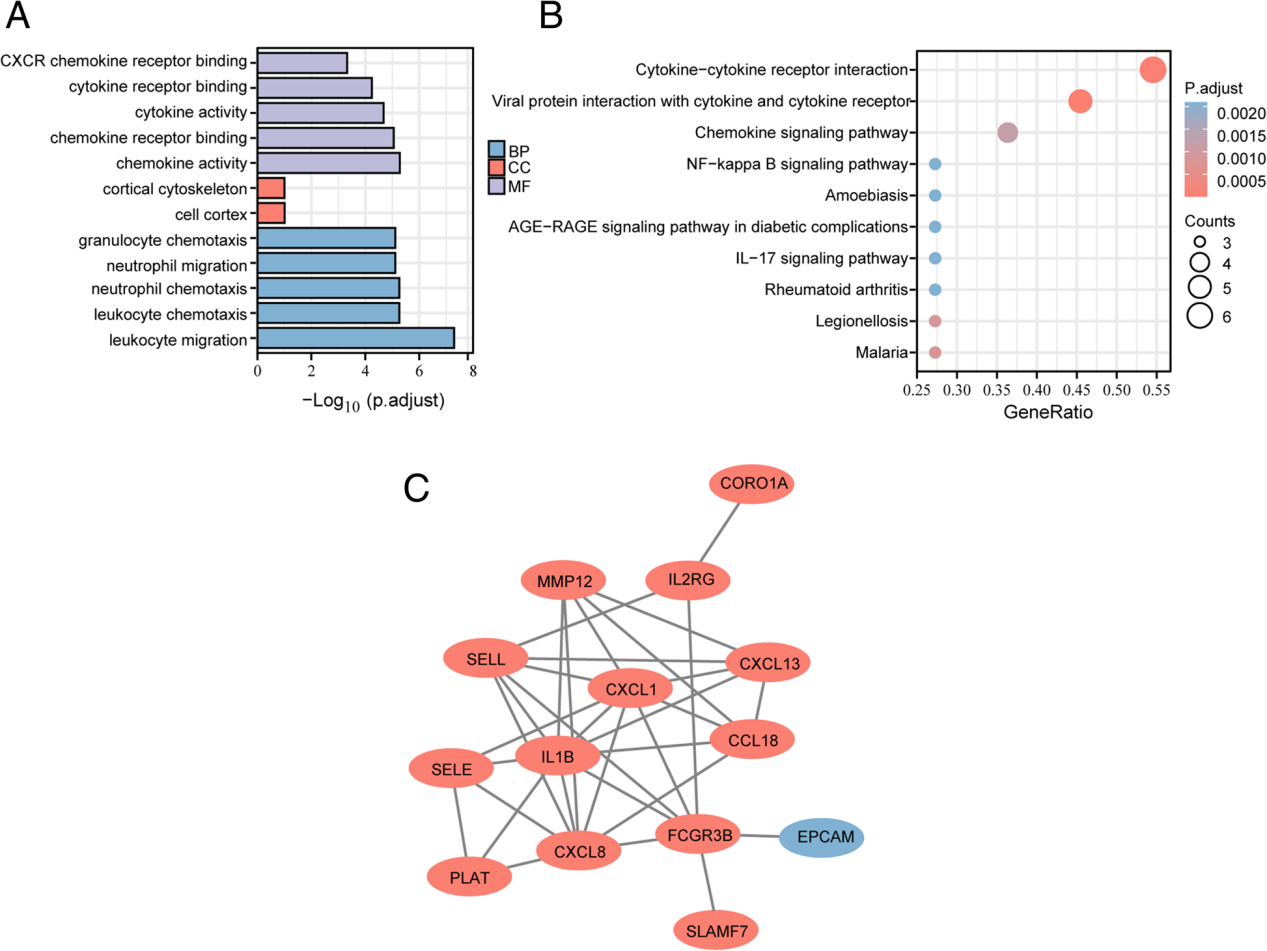
Enrichment analysis and PPI network of common DEGs. **A**. GO enrichment analysis of common DEGs. Top 5 of BP and MF are shown. **B**. KEGG pathway of common DEGs. Top 10 are shown. **C**. PPI network of common DEGs contains 14 nodes and 33 interactions. Orange represents upregulated genes, and blue represents downregulated genes

Analysis of GO and KEGG pathway enrichment were performed to explore the functions and pathways of the 18 common DEGs. From GO analysis, we found that these common DEGs enrich in neutrophil chemotaxis (P.adjust = 5.48784E-06), neutrophil migration (P.adjust = 5.48784E-06), chemokine activity (P.adjust = 5.25019E-06), chemokine receptor binding (P.adjust = 8.84745E-06), CXCR chemokine receptor binding (P.adjust = 0.000475041) (Fig. 3A). According to KEGG pathway, the four major enrichment pathways are Cytokine-cytokine receptor interaction (P.adjust = 2.95035E-05), Chemokine signaling pathway (P.adjust = 0.001183363), IL-17 signaling pathway (P.adjust = 0.002095938), NF-kappa B (NF-κB) signaling pathway (P.adjust = 0.002095938) (Fig. 3B). These results strongly suggest that neutrophil chemotaxis, inflammatory factors, and autoimmunity are primarily responsible for the comorbidity of PSO and PD.

### PPI network construction and identification of hub genes

**Fig. 4:**
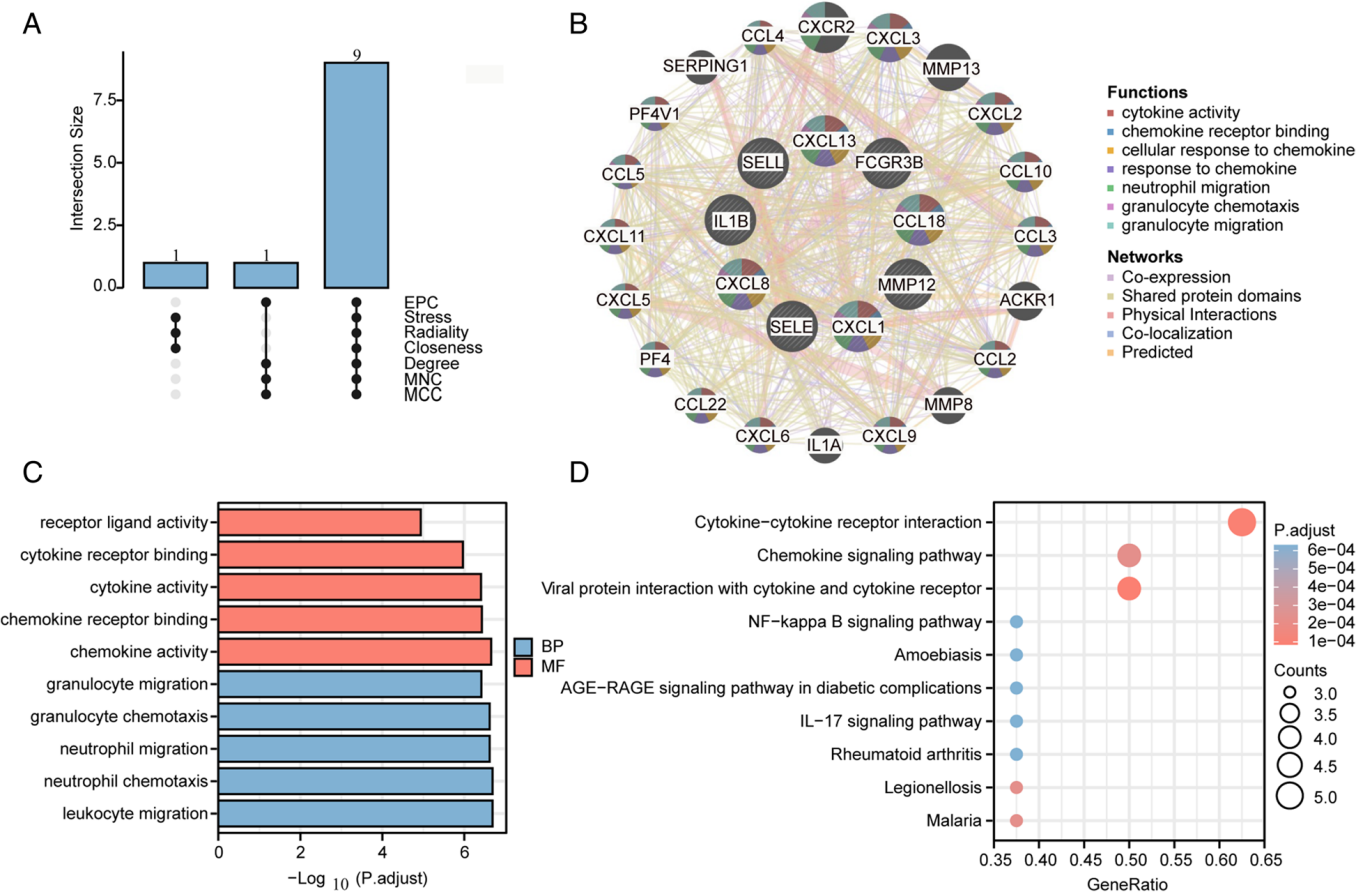
Identification and analysis of hub genes. **A**. The upset diagram shows that there are 9 overlapping hub genes based on seven algorithms. **B**. Hub genes and their co-expression genes network. **C**. GO enrichment analysis of hub genes. Top 5 of BP and MF are shown, and there is no CC. **D**. KEGG pathway of hub genes. Top 10 are shown

Cytoscape was used to build PPI networks containing 14 nodes and 33 interactions for the common DEGs (Fig. 3C). The hub genes were evaluated and selected by seven common algorithms of Cytohubba (Supplementary Table 2). 9 hub genes were acquired at the intersection of the Upset diagram, including IL1B, CXCL1, CXCL8, MMP12, CCL18, SELL, CXCL13, FCGR3B, and SELE (Fig. 4A). The details of their roles were searched in the HGNC database (Supplementary Table 3).

### Co-expression and enrichment analysis of hub genes

According to the co-expression network of hub genes, we obtained the top 7 related functions and different weight of interaction, including co-expression of 46.46%, shared protein domains 21.24%, physical interactions of 14.35%, co-localization of 13.92%, predicted of 4.03% (Fig. 4B). The GO analysis revealed that these genes mostly regulate neutrophil chemotaxis (P.adjust = 2.04559E-07), neutrophil migration (P.adjust = 2.39494E-07), chemokine activity (P.adjust = 2.19922E-07), chemokine receptor binding (P.adjust = 3.72607E-07), cytokine activity (P.adjust = 3.89078E-07), cytokine receptor binding (P.adjust = 1.08135E-06), receptor ligand activity (P.adjust = 1.14917E-05) (Fig. 4C). Meanwhile, The KEGG pathway of these hub genes was revealed, including Cytokine-cytokine receptor interaction (P.adjust = 9.16908E-05), Chemokine signaling pathway (P.adjust = 0.000229325), IL-17 signaling pathway (P.adjust = 0.000631912), NF-κBsignaling pathway (P.adjust = 0.000631912). These results also emphasized the important role of neutrophil chemotaxis, inflammatory factors, and autoimmunity in these two diseases (Fig. 4D).

### Exploration of common miRNAs in PSO and PD

**Fig. 5:**
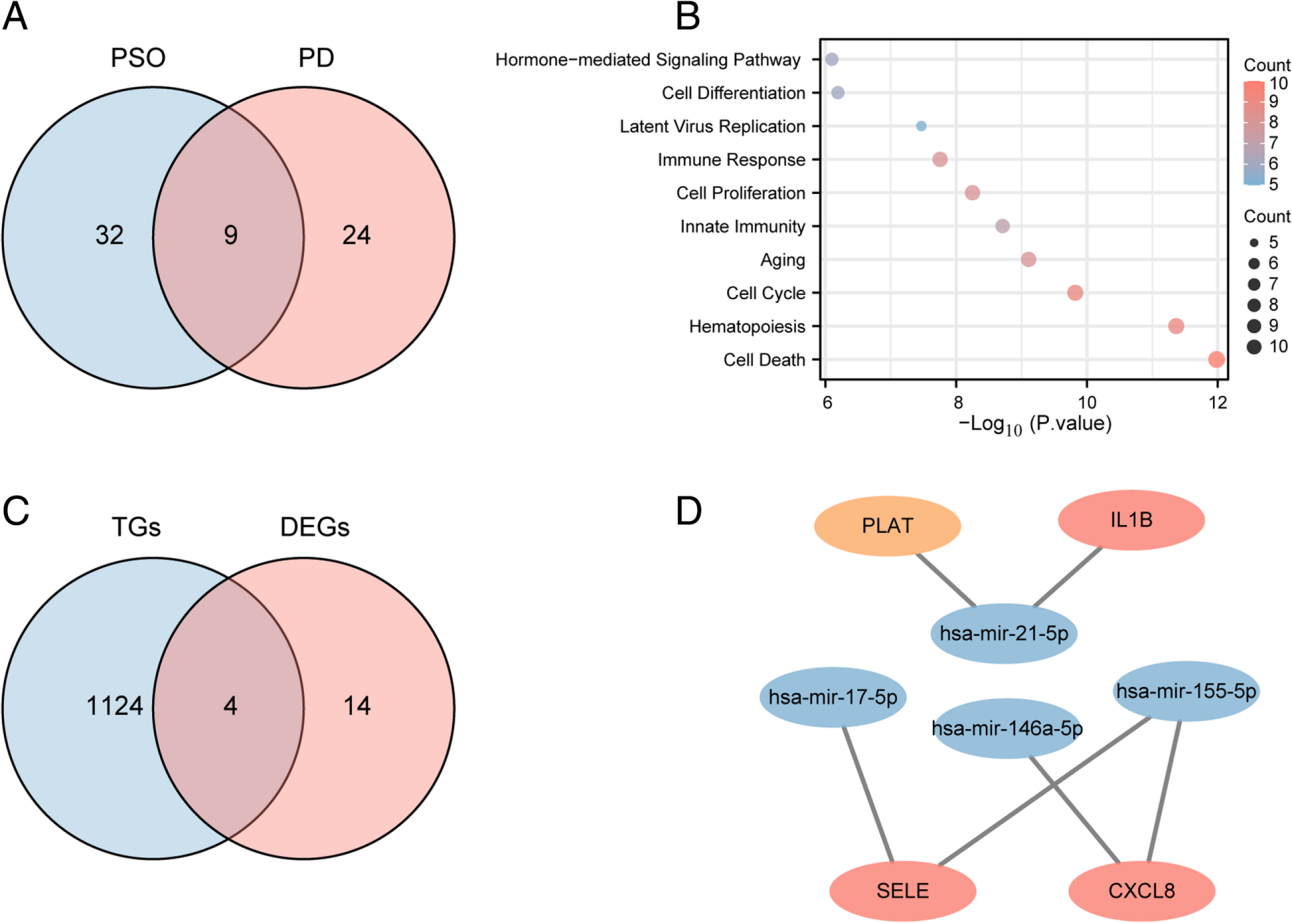
Exploration of miRNAs. **A**.9 overlapping miRNA in psoriasis and periodontitis. **B**. Functions of common miRNAs, Top 10 are shown. **C**. 4 overlapping genes between target genes of miRNAs and common DEGs. **D**. The miRNAs-mRNAs network. Blue represents miRNAs, pink represents hub genes, and yellow represents common DEGS

Totally, 41 miRNAs related to PSO and 33 miRNAs related to PD were identified via the HMDD database. There were 9 common miRNAs between PSO and PD shows in the Veen diagram, including hsa-let-7a-5p, hsa-mir-100-5p, hsa-mir-125b-5p, hsa-mir-130a-3p, hsa-mir-146a-5p, hsa-mir-155-5p, hsa-mir-17-5p, hsa-mir-19b-1-5p, hsa-mir-21-5p (Fig. 5A). These miRNAs involved in Cell Cycle (*P*.value = 1.35E-10), Aging (*P*.value = 7.06E-10), Granulopoiesis (*P*.value = 1.34E-09), Innate Immunity (*P*.value = 1.78E-09), Cell Proliferation (*P*.value = 5.10E-09), Immune Response (*P*.value = 1.60E-08), Latent Virus Replication (*P*.value g = 3.21E-08) (Fig. 5B).

### The common miRNAs- mRNAs network

A total of 1128 target genes were obtained via miRTarbase. 4 important genes were found in both 1128 target genes and 18 common DEGs, including CXCL8, SELE, IL1B, PLAT. The first three genes of them are hub genes (Fig. 5C). Then, the miRNAs-mRNAs network was established, showing the relationship between 4 miRNAs and 4 mRNAs (Fig. 5D).

### TFs prediction of hub genes

**Fig. 6:**
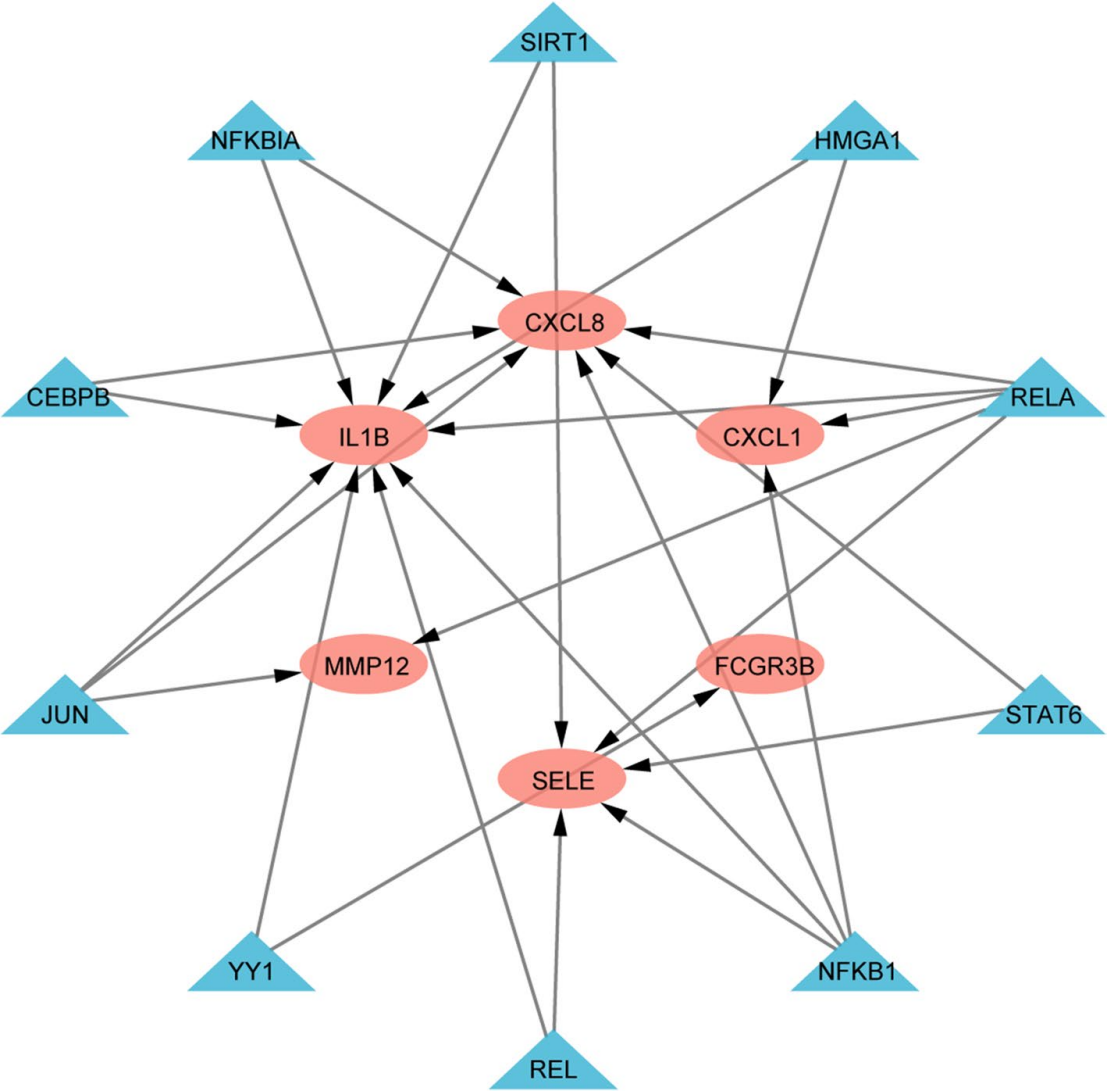
Regulatory network of TFs. pink represents related hub genes, and green represents TFs

On the basis of the TTRUST database, 10 TFs were obtained that regulates the hub genes. Details such as description, p value have been recorded (Supplementary Table 4). The TFs–Hub genes network was constructed, including 16 nodes and 26 edges (Fig. 6).

## Discussion

In this study, we screened 18 common DEGs and 9 common hub genes through bioinformatics analysis, further identified 9 common miRNAs based on experimental evidence and 10 common TFs in both PSO and PD. Meanwhile, we identified signaling pathways that are closely related to these genes and miRNAs, especially the top 10 pathways with significant differences ranked by P.adjust value, which will be the key point of our discussion about comorbidity mechanism in PSO and PD next.

The pathogenesis of PSO and PD is relatively complex, and new biomarkers are still being reported. Here, we summarized the pathogenesis of PSO and PD. The main pathogenesis of PSO is: (i) The external environment stimulates keratinocytes to produce antibacterial peptides, growth factors, chemokines, triggering the initial response of immune system to produce pro-inflammatory factors. (ii) Helper T cell type 17 (Th17) produces IL-17 and IL-22 by activating pathways such as JAK-STAT and NF-κB signaling pathways to amplify inflammation, causing excessive proliferation of keratinocytes and dynamic inflammatory response [1, 4, 25, 26].The main pathogenesis of PD: (i) Pathogens activate NF-κB signaling pathways to produce pro-inflammatory factors such as TNF, IL1B, IL-17and IL-18, enhancing the expression of the receptor activator of NF-κB ligands (RANKL) in neutrophils and osteoblasts, driving osteoclast maturation. The imbalance of RANKL and osteoprotegerin (OPG) can directly stimulates osteoclastogenesis. (ii) Immune cells release matrix metalloproteinases and reactive oxygen species to destroy the extracellular matrix in periodontal tissue. (iii) Increased vascular permeability can allow pro-inflammatory mediators and antimicrobial peptides to enter the bloodstream, causing distal inflammation [27–31]. Of course, there are also studies that specifically review the molecular mechanisms of comorbidities of PSO and PD from the aspects of epidemiology, genetics, microbiology and immunology. They pointed out that IL-17 signaling pathway, which plays a central role in PSO, is also particularly important during bone loss of PD [32, 33].

Consistent with the above researches, our results also reveal the important roles of IL-17 signaling pathway, Cytokine-cytokine receptor interaction, Chemokine signaling pathway, NF-κB signaling pathway from the perspective of bioinformatics in PSO and PD. Moreover, the remaining top 10 signaling pathways can be divided into two categories, one is the response of viruses, malaria parasites, legionella, and amoebas after infection, showing the role of host defense pathways in PSO and PD [34, 35]. The other category is disease-related pathways: rheumatoid arthritis and AGE-RAGE signaling pathway in diabetic complications. It suggests that PSO and PD have similar molecular mechanisms with rheumatoid arthritis and diabetes’ complications. It is worth thinking that the AGE-RAGE signaling pathway has been studied deeply in the complications of diabetes, including diabetic nephropathy [36], osteoporosis [37] and cardiovascular disease [38].Meanwhile, The AGE-RAGE signaling pathway in diabetic complications has been included as a separate signaling pathway in the KEGG database, which shows the significance of related research. At present, there are no mechanisms related to the complications of PSO or PD that are separately included in the KEGG database, but the oral health of PSO patients or the skin health of PD patients need to be paid attention to [39–41]. Our research can pave the way for studying the mechanisms of oral health in PSO patients or skin health in PD patients, in order to explore pathways similar to AGE-RAGE signaling pathway in diabetic complications to explain as much as possible the mechanism behind multiple complications in PSO and PD.

Another point worth emphasizing is that we select experimentally validated data as the basis for both screening common miRNAs and screening genes that interact with miRNAs. The HMDD database collects proven miRNA data from diseases [42], and in the miRTarbase database, we limit the screening criteria to experimentally verified interactions [43]. It can increase the credibility of our study compared to databases that make predictions only by sequence combination. The results of function enrichment analysis of common miRNAs also indicate the important roles of immune Response, innate Immunity and inflammation. Also, there are still some limitations in our research:(i) We did not have further experimental verification, even if we try to choose experimentally-based databases during our analysis. (ii) Due to the limitations of public data set sample size and sequencing platform, the data sets we selected are all local tissue samples of diseases rather than blood samples. This may affect the representativeness of our findings. (iii) In our study, the total sample size for PSO was less than for PD, and this imbalance in sample size may also affect the discovery of DEGs. (iv)All findings depend on transcription levels. There was no basic information of patients including age, sex, comorbidities or smoking habits and so on. (v) When selecting the dataset of periodontitis, we only focused on the size of the samples, while ignoring the overlap of the samples. Strictly speaking, they cannot be used as mutual verification, and more datasets should be selected for verification in subsequent studies. Therefore, the generalization of our findings is limited.

In summary, our study is the first to directly identify common DEGs, hub genes, miRNAs, TFs, and their enriched pathways in PSO and PD from the perspective of bioinformatics. These results may help to reveal the comorbidity mechanisms of PSO and PD, although there is no further experimental verification. Meanwhile, it can provide a theoretical framework and basis for future research. Next, well-designed clinical prospective studies based on our data are necessary to directly detect the changes occurring in patients with both PSO and PD. In this process, confounding factors such as smoking and drinking can be adjusted to further improve the reliability and clinical promotion value of the study.

## Conclusion

Our study revealed possible comorbidity mechanisms in PSO and PD from the perspective of bioinformatics tentatively. The data can present new insight for joint prevention and treatment of PSO and PD, as well as provide data support for further prospective studies.

## Materials and methods

### Data source

The GEO database (http://www.ncbi.nlm.nih.gov/geo) contains millions of microarray datasets and high-throughput sequences submitted by researchers worldwide. We used the key word “Psoriasis” or “Periodontitis” to search gene expression datasets. Inclusion and exclusion criteria:(i) the test specimens should be Homo sapiens (ii) the gene expression profiles should include cases as well as controls. (iii) The samples of both diseases should be local pathological tissues. The PSO are skin tissues, and the PD are gum tissues. (iv) Preferentially select the datasets with large sample size (v) The sequencing platform should be consistent. (vi) Patients receiving clinical intervention are excluded. As a result, the GEO datasets: GSE30999, GSE14905, GSE16134, and GSE10334 were selected. For GSE30999 and GSE14095, we used dataset full soft file for further analysis. For GSE16134, GSE10334, we matched the gene symbols and probes based on platform file and performed log2 transformed to acquire gene matrix for the final analysis,

### Identification of DEGs

R package “limma” was used to identify DEGs. R package “Impute” was used to replenish the missing expression data. Multiple probes corresponding to a single gene symbol were averaged and probes without a corresponding gene symbol were removed. The criteria of DEGs are and |logFC (fold change)|≥ 1 and adjusted P-value < 0.05. The Venn diagram drew by R package “ggplot2” was used to obtain their common DEGs. R version is 3.6.3.

### Enrichment analysis of common DEGs

R Package “clusterProfiler” was used to perform GO and KEGG pathway enrichment analysis. The GO analysis contains three terms: biological process, cellular component, and molecular function. R Package “ggplot2” was used for visualization.

### PPI network construction and hub genes selection

String (http://string-db.org) was used to explore the complex regulatory relationships among proteins of interest. The combined score over 0.4 was set as statistically significant. Seven different algorithms of Cytohubba in Cytoscape were used to explore hub genes.

### Analysis of hub genes

R Package “clusterProfiler” was used to perform GO and KEGG pathway enrichment analysis same as before. GeneMANIA (http://www.genemania.org) is an effective tool for identifying the interconnectedness of gene sets, through which we established the co-expression network of these hub genes.

### Identification of common miRNAs in PSO and PD

The miRNAs exert gene regulation by degrading mRNA or inhibiting its function. Here, we searched for miRNAs associated with two diseases based on the HMDD database (http://www.cuilab.cn/hmdd), which is full of experimental evidence related to miRNAs and disease. The PSO-associated miRNA and the PD-associated miRNA were obtained and intersected, in addition, the mature miRNA was searched in the miRDB database (http://mirdb.org) to conduct further analysis. TAM 2.0 (http://www.lirmed.com/tam2) was used to perform miRNA function analysis. The terms ranked by *p*-values and *p*-value < 0.05 were identified as significant.

### The common miRNAs-mRNA network construction

MiRTarbase is an experimentally validated miRNA-target interactions database (https://mirtarbase.cuhk.edu.cn), which included amounts of miRNAs and target genes supported by experimental evidence. Taking intersection of common DEGs and target genes of predicted consensus miRNAs in PSO and PD. The miRNAs–mRNAs regulated network was established.

### Prediction of TFs

TRRUST (https://www.grnpedia.org/trrust) includes the target genes of common TFs and the regulatory relationship between TFs, which is usually used for the prediction of transcriptional regulatory networks. A total of 8,444 and 6,552 TFs target regulatory relationships is recorded in this database. TFs that regulate the hub genes were searched from TRRUST, and *P*.value < 0.05 was included in the analysis.

### Supplementary Information

**Additional file 1:** **Supplementary Table 1.** The datasets used for analysis. **Supplementary Table 2.** The top 10 hub genes rank in Cytohubba. **Supplementary Table 3.** The details of the hub genes. **Supplementary Table 4.** The key transcriptional factors (TFs) of hub genes.

## Data Availability

There is no new datasets and materials.

## Abbreviations

PSO: Psoriasis
PD: Periodontitis
DEGs: Differentially expressed genes
FC: Fold change
TFs: Transcription factors
