# Correction: Exploration of the underlying comorbidity mechanism in psoriasis and periodontitis: a bioinformatics analysis

**DOI:** 10.1186/s41065-024-00315-1

**Published:** 2024-04-04

**Authors:** Hao Lei, Xin Chen, Ziyang Wang, Zixuan Xing, Wenqian Du, Ruimin Bai, Ke He, Wen Zhang, Yan Wang, Yan Zheng

**Affiliations:** 1 Department of Dermatology, the First Affiliated Hospital of Xi’an Jiaotong University, Xi’an, 710061 China; 2 State Key Laboratory of Military Stomatology & National Clinical Research Center for Oral Diseases & Shaanxi Clinical Research Center for Oral Diseases, Department of Orthodontics, School of Stomatology, The Fourth Military Medical University, Xi’an, 710032 China; 3 Department of Medicine, Xi’an Jiaotong University, Xi’an, 710061 China

**Correction: Hereditas 160, 7 (2023).**

10.1186/s41065-023-00266-z

Following publication of the original article [1], the authors reported an error in selection of periodontitis datasets. The two datasets used for differential gene analysis of periodontitis had overlapping samples. To be strict, they cannot be used as mutual verification. So, the authors clarified the limitations of the overlapping datasets in discussion part. It was added as limitation five: (V) When selecting the dataset of periodontitis, we only focused on the size of the samples, while ignoring the overlap of the samples. Strictly speaking, they cannot be used as mutual verification, and more datasets should be selected for verification in subsequent studies. The original article [1] has been updated.
