# Supplementary material for: Exploration of the underlying comorbidity mechanism in psoriasis and periodontitis: a bioinformatics analysis
Source: Hereditas. 2023 Feb 10;160:7. doi: 10.1186/s41065-023-00266-z (PMC9912623; doi:10.1186/s41065-023-00266-z)
Supplement: Supplementary file 1 — Additional file 1: Supplementary Table 1. The datasets used for analysis. Supplementary Table 2. The top 10 hub genes rank in Cytohubba. Supplementary Table 3. The details of the hub genes. Supplementary Table 4. The key transcriptional factors (TFs) of hub genes. [file 41065_2023_266_MOESM1_ESM.docx]

**The Supplementary information of this study**

**Supplementary Table 1: The datasets used for analysis.**

| **Disease** | **Series** | **Platforms** | **Case** | **Control** | **Total** |
| --- | --- | --- | --- | --- | --- |
| Psoriasis | GSE30999 | GPL570 | 85 | 85 | 170 |
|  | GSE14905 | GPL570 | 33 | 28 | 61 |
| Periodontitis | GSE16134 | GPL570 | 241 | 69 | 310 |
|  | GSE10334 | GPL570 | 183 | 64 | 247 |

**Supplementary Table2: The top 10 hub genes rank in Cytohubba.**

| **MCC** | **MNC** | **Degree** | **Closeness** | **Radiality** | **Stress** | **EPC** |
| --- | --- | --- | --- | --- | --- | --- |
| IL1B | IL1B | IL1B | IL1B | IL1B | FCGR3B | IL1B |
| CXCL1 | CXCL1 | CXCL1 | CXCL1 | CXCL1 | IL1B | CXCL1 |
| CXCL8 | CXCL8 | CXCL8 | CXCL8 | CXCL8 | SELL | CXCL8 |
| MMP12 | SELL | FCGR3B | FCGR3B | FCGR3B | CXCL8 | SELL |
| CCL18 | MMP12 | SELL | SELL | SELL | IL2RG | FCGR3B |
| SELL | CCL18 | MMP12 | CXCL13 | CXCL13 | CXCL1 | CXCL13 |
| CXCL13 | CXCL13 | CCL18 | MMP12 | MMP12 | CXCL13 | CCL18 |
| FCGR3B | FCGR3B | CXCL13 | CCL18 | CCL18 | MMP12 | MMP12 |
| SELE | SELE | SELE | SELE | SELE | CCL18 | SELE |
| PLAT | PLAT | PLAT | IL2RG | IL2RG | SELE | PLAT |

**Supplementary Table3: The details of the hub genes.**

| **No.** | **Genes** | **Full name** | **Functions** |
| --- | --- | --- | --- |
| 1 | IL1B | interleukin 1 beta | angiogenesis, cellular response to biotic stimulus, cellular response to lipopolysaccharide, cellular response to molecule of bacterial origin, cytokine receptor binding, etc. |
| 2 | CXCL1 | C-X-C motif chemokine ligand 1 | antimicrobial humoral response, cell chemotaxis, cellular response to biotic stimulus, cellular response to chemokine, cellular response to molecule of bacterial origin, etc. |
| 3 | CXCL8 | C-X-C motif chemokine ligand 8 | angiogenesis, antimicrobial humoral response, cell chemotaxis, cellular response to biotic stimulus, cellular response to chemokine, cellular response to molecule of bacterial origin,etc. |
| 4 | MMP12 | matrix metallopeptidase 12 | collagen metabolic process. |
| 5 | CCL18 | C-C motif chemokine ligand 18 | cell chemotaxis, cellular response to chemokine, chemokine receptor binding, cytokine activity, cytokine receptor binding, ERK1 and ERK2 cascade, etc. |
| 6 | SELL | selectin L | cellular extravasation, glycosaminoglycan binding, leukocyte cell-cell adhesion, leukocyte migration, sulfur compound binding. |
| 7 | CXCL13 | C-X-C motif chemokine ligand 13 | angiogenesis, antimicrobial humoral response, CCR chemokine receptor binding, cell chemotaxis, cell-cell adhesion mediated by integrin, cellular calcium ion homeostasis,etc. |
| 8 | FCGR3B | Fc gamma receptor IIIb | capture immune complexes in the peripheral circulation. |
| 9 | SELE | selectin E | cellular extravasation, leukocyte cell-cell adhesion, leukocyte migration, positive regulation of cell adhesion, positive regulation of cell-cell adhesion, etc. |

**Supplementary Table4: The key transcriptional factors（TFs）of hub genes.**

| **Key TF** | **Description** | **P value** | **Genes** |
| --- | --- | --- | --- |
| RELA | v-rel reticuloendotheliosis viral oncogene homolog A (avian) | 1.19E-07 | CXCL8,IL1B,MMP12,SELE,CXCL1 |
| NFKB1 | nuclear factor of kappa light polypeptide gene enhancer in B-cells 1 | 7.70E-06 | IL1B,CXCL8,SELE,CXCL1 |
| HMGA1 | high mobility group AT-hook 1 | 2.74E-05 | CXCL1,IL1B |
| NFKBIA | nuclear factor of kappa light polypeptide gene enhancer in B-cells inhibitor, alpha | 3.44E-05 | IL1B,CXCL8 |
| JUN | jun proto-oncogene | 3.91E-05 | IL1B,CXCL8,MMP12 |
| REL | v-rel reticuloendotheliosis viral oncogene homolog (avian) | 4.65E-05 | IL1B,SELE |
| STAT6 | signal transducer and activator of transcription 6, interleukin-4 induced | 0.000126 | SELE,CXCL8 |
| SIRT1 | sirtuin 1 | 0.000226 | IL1B,SELE |
| CEBPB | CCAAT/enhancer binding protein (C/EBP), beta | 0.000353 | CXCL8,IL1B |
| YY1 | YY1 transcription factor | 0.00081 | IL1B,FCGR3B |
